# Genomic Alterations Correlated to Trastuzumab Resistance and Clinical Outcomes in HER2+/HR- Breast Cancers of Patients Living in Northwestern China

**DOI:** 10.7150/jca.84832

**Published:** 2024-06-17

**Authors:** Gang Ma, Binliang Huo, Yanwei Shen, Xulong Zhu, Chong Cheng, Wensheng Li, Wei Cao, Jianhui Li

**Affiliations:** 1 Department of Surgical Oncology, Shaanxi Provincial People's Hospital, Xi'an, China.; 2 Department of Pathology, Shaanxi Provincial People's Hospital, Xi'an, China.

**Keywords:** Genomic profiling, Breast cancer, HER2 positive, Trastuzumab resistance

## Abstract

Anti-HER2 therapy has significantly improved the survival rates of patients with HER2+ breast cancer. However, a subset of these patients eventually experience treatment failure, and the underlying genetic mechanisms remain largely unexplored. This underscores the need to investigate the genomic heterogeneity of HER2+ breast cancer. In this study, we focus on HER2+/HR- breast cancer, as it differs from HER2+/HR+ breast cancer in terms of genetic and biological characteristics. We performed gene-targeted genome sequencing on 45 HER2+/HR- breast cancer samples and identified 650 mutations across 268 cancer-related genes. TP53 (71.1%) and PIK3CA (35.6%) were the most frequently mutated genes in our sample. Additionally, ERBB2 (77.8%), CDK12 (42.2%), and MYC (11.1%) exhibited a high frequency of copy number amplifications (CNAs). Comparative analysis with two other HER2+/HR- breast cancer cohorts revealed that our cohort had higher genetic variation rates in ARID1A, PKHD1, PTPN13, FANCA, SETD2, BRCA2, BLM, STAG2, FAT1, TOP2A, POLE, ATM, KMT2B, FGFR4, and EPAS1. Notably, in our cohort, NF1 and ATM mutations were more prevalent in trastuzumab-resistant patients (NF1, p=0.016; ATM, p=0.006) and were associated with primary trastuzumab resistance (NF1, p=0.042; ATM, p=0.021). Moreover, patients with NF1 mutations (p=0.009) and high histological grades (p=0.028) were more likely to experience early relapse. Ultimately, we identified a unique cancer-related gene mutation profile and a subset of genes associated with primary resistance to trastuzumab and RFS in patients with HER2+/HR- breast cancer in Northwest China. These findings could lay the groundwork for future studies aimed at elucidating the mechanisms of resistance to trastuzumab and improving HER2-targeted treatment strategies.

## Introduction

Breast cancer is the most prevalent cancer among women. There are approximately 306,000 new cases of female breast cancer reported in China annually, leading to approximately 71,700 deaths 1. Roughly a quarter of patients with breast cancer exhibit *HER2* (also known as *ERBB2*) gene amplification and therefore require anti-HER2 therapy 2. The introduction of anti-HER2 medications, including monoclonal antibodies, tyrosine kinase inhibitors, and antibody-drug conjugates, has significantly improved survival rates for patients with HER2-positive breast cancer 3. However, approximately 20% of patients with HER2-positive breast cancer do not respond to trastuzumab initially, and about 70% of patients with metastatic disease who receive trastuzumab are resistant to treatment 4-7, underscoring the importance of investigating the genetic intricacies of HER2-positive breast cancer due to its heterogeneity. It is recognized that HER2-positive/hormone receptor-positive (hereinafter referred to as HER2+/HR+) and HER2-positive/hormone receptor-negative (hereinafter referred to as HER2+/HR-) breast cancers are biologically and genetically different 8. To eliminate the effect of the estrogen receptor pathway on HER2-positive breast cancer, this study focuses on HER2+/HR- breast cancer. We employed a gene-targeted genome sequencing approach, analyzing 425 cancer genomes, to explore the relationship between known cancer-associated genes and HER2+/HR- breast cancer in northwestern China.

## Materials and Methods

### Patients and sample collection

Forty-five women diagnosed with HER2+/HR- breast cancer at Shaanxi Province Hospital between January 2016 and December 2018 were included in this study. Clinical and pathological data were collected from each patient, including age, menopausal status, TNM stage based on the AJCC 7^th^ edition 9, lymph node status, estrogen receptor (ER) status, progesterone receptor (PR) status, HER2 status, and Ki67 index. Two experienced pathologists determined the immunohistological results. When the HER2 result was 2+, FISH detection was performed to confirm the HER2 status 10. All patients underwent surgery, followed by standard adjuvant regimens containing doxorubicin and paclitaxel. Additionally, all patients received 1-year standard trastuzumab treatment and were followed-up for a median of 36 months. This study was approved by the Ethics Committee of Shaanxi Provincial People's Hospital and was conducted in accordance with the principles of the Declaration of Helsinki.

### Sequencing and bioinformatics analysis

Formalin-fixed, paraffin-embedded (FFPE) tumor tissue blocks of 45 patients with HER2+/HR- breast cancer were obtained from the Pathology Department of Shaanxi Province People's Hospital. Pathologists verified the tumor purity for each sample. The blocks were de-paraffinized using xylene and genomic DNA was extracted using the QIAamp DNA FFPE Tissue kit (Qiagen). The extracted genomic DNA was quantified with Nanodrop2000 (Thermo Fisher Scientific, Waltham, MA), and further assessed using a dsDNA HS Assay kit on a Qubit 3.0 Fluorometer (Life Technologies, Carlsbad, CA). Sequencing libraries were prepared with the KAPA Hyper Prep kit (KAPA Biosystems, Wilmington, MA, USA), following previously described methods 11. Hybridization-based targeted gene capture utilized the Geneseeq pan-cancer gene panel (425 cancer-relevant genes, Supplementary Table 1), and the targeted libraries were sequenced on an Illumina HiSeq4000 NGS platform, achieving a mean coverage depth exceeding 300×. Quality trimming was performed with Trimmomatic 12 to remove bases of quality lower than 15 or N bases, followed by mapping to the hg19 genome (Human Genome version 19) using the Burrows-Wheeler Aligner. Local realignments around indels and base quality control, along with germline mutation detection, were conducted using the Genome Analysis Toolkit (GATK3.4.0). Somatic SNPs and indels were identified with VarScan2 13 and HaplotypeCaller/UnifiedGenotyper in GATK. The mutant allele frequency (MAF) cut-off was set at 0.1% for samples, and a minimum of three unique mutant reads was needed. Common SNPs were filtered out using dbSNP (v137) and the 1000 Genome database. Gene fusions were detected with FACTERA software 14. The copy number variations (CNVs) were analyzed using ADTEx, with a log2 ratio cut-off of 2.0 for copy number gain in samples.

### Statistical methods

Categorical variables are represented by frequency and percentage, while quantitative variables are presented as mean ± SEAM. To compare categorical variables, the chi-square or Fisher's exact test was employed, and logistic regression analysis was utilized to examine potential predictive factors for trastuzumab resistance. The log-rank test and Cox regression analysis were applied to assess prognostic factors for recurrence-free survival (RFS) (SPSS 18.1.0 for Windows, SPSS Inc., Chicago, IL, USA). Survival curves were generated with GraphPad Prism8. All statistical tests were two-sided, with statistical significance established at *P*< 0.05.

## Results

### Clinical characteristics

We performed specific gene sequencing on FFPE tumor samples from 45 patients with HER2+/HR- breast cancer, whose detailed clinical characteristics are presented in Table 1. These patients were classified based on their menopausal status, TNM stage, lymph node stage, histological grade, Ki67 index, and follow-up outcomes. The median age of the cohort was 53 years with a range of 26-80 years. Among the 45 patients, 62.2% were postmenopausal, 71.1% were at TNM II stage, 75.5% had N1 lymph node involvement, 37.8% were grade 3, and 57.8% had a Ki67 index over 30%. About 33.3% (15/45) experienced recurrence within the median follow-up period of 36 months, with 66.7% (10/15) of these recurrences occurring within 24 months post-operation.

### The mutation spectrum of northwest Chinese patients with HER2+/HR- breast cancer

**Figure 1:**
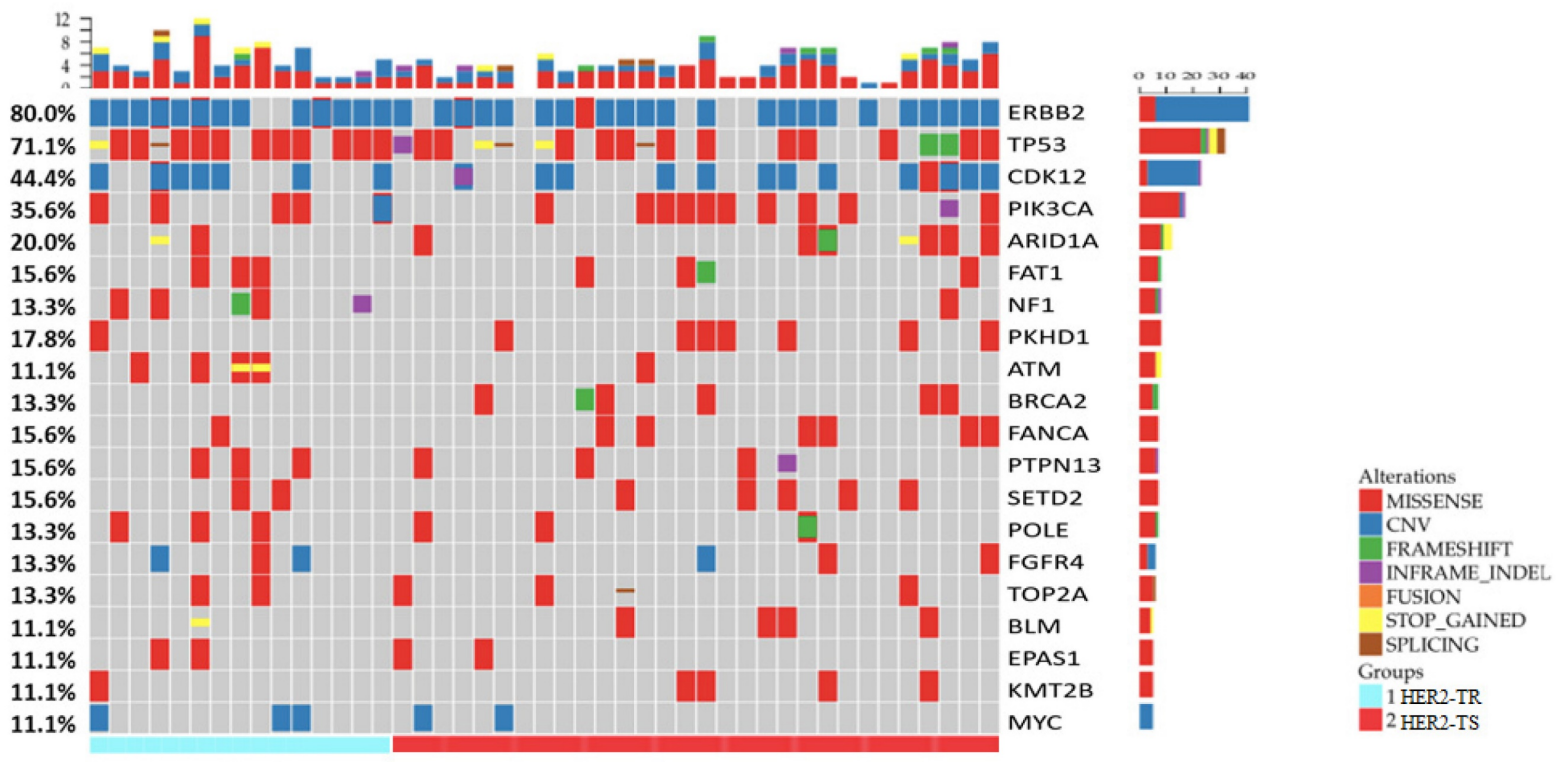
** Summary of the somatic mutations and Indels of all 45 patients with HER2+/HR- breast cancer in Northwestern China.** (HER2-TR = HER2+/HR- breast cancer with primary trastuzumab-resistant status, HER2-TS = HER2+/HR- breast cancer with tastuzumab sensitive status).

In total, 650 mutations were identified, including 458 single nucleotide variations (SNVs), 38 insertion-deletion variants with 25 frameshift variants (indels), 22 nonsense variants, 21 splice site variants, 96 copy number amplifications (CNA), and 13 structural variations (SV), spanning 268 genes out of the 425 genes listed. Among these 268 genes, 17 were frequently mutated, exhibiting mutation rates greater than 10%. These included: *TP53* (71.1%), *PIK3CA* (35.6%), *ARID1A* (20.0%), *PKHD1* (17.8%), *FAT1* (15.6%), *FANCA* (15.6%), *PTPN13* (15.6%), *SETD2* (15.6%), *NF1* (13.3%), *POLE* (13.3%), *TOP2A* (13.3%), BRCA2 (13.3%), *ATM* (11.1%), *BLM* (11.1%), *EPAS1* (11.1%), *KMT2B* (11.1%), and *ERBB2* (11.1%). In addition, genes with high-level CNA exceeding 10% included *ERBB2* (77.8%, fold change 2-25), *CDK12* (42.2%, fold change 2-9), and *MYC* (11.1%, fold change 1.6-24). A summary of the gene alterations in our cohort is depicted in Figure 1.

**Figure 2:**
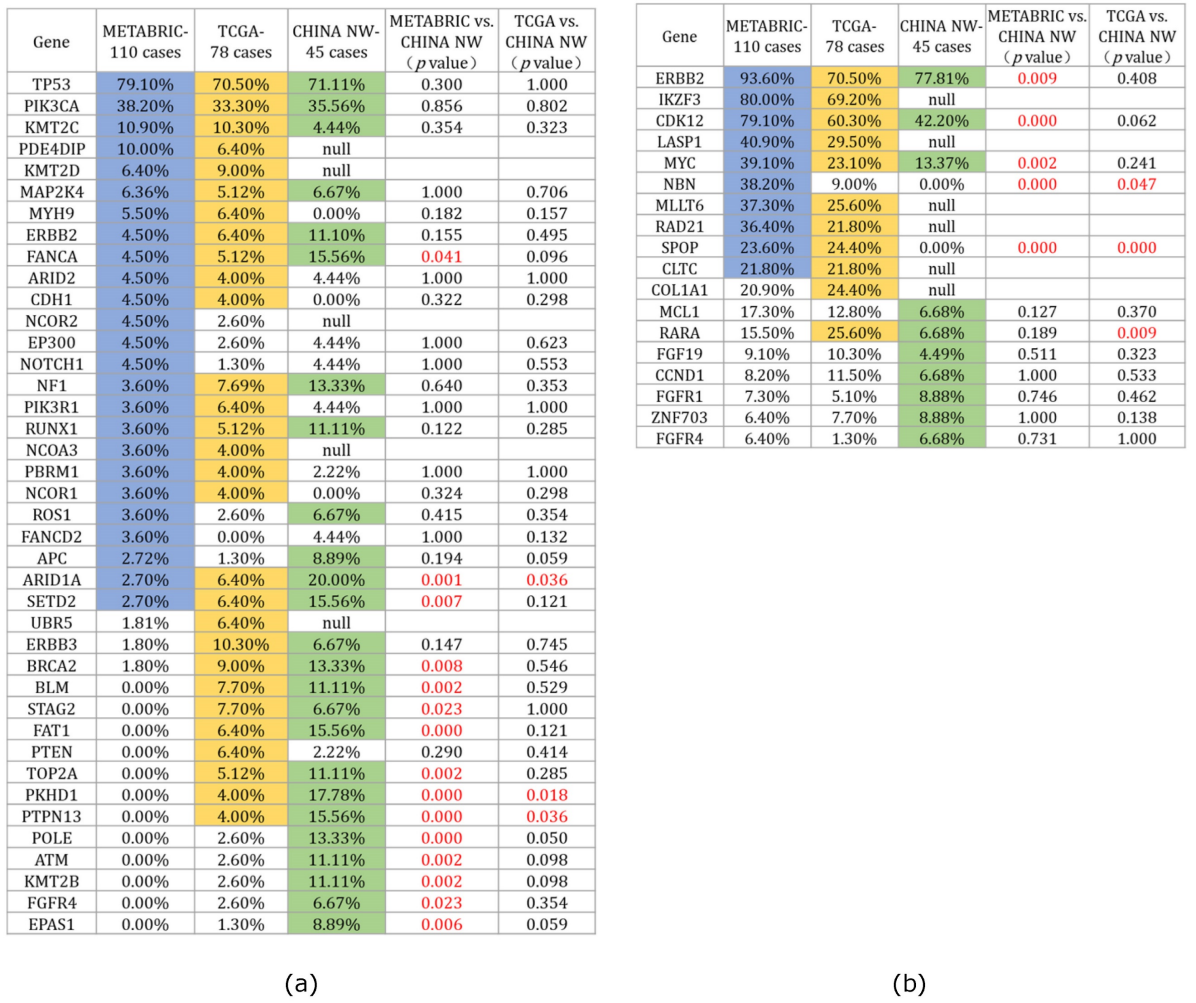
** Comparison of gene mutations and CNAs between our cohort and the two published HER2+/HR- breast cancer cohorts (TCGA PanCancer Atlas and METABRIC):** (a) Comparison of the top twenty mutated genes among the three cohorts (the blue, yellow, and green colors represent the top twenty mutated genes of the three cohorts individually); (b) Comparison of top ten CNAs among the three cohorts (the blue, yellow, and green colors indicate the top ten CNAs of the three cohorts individually).

To investigate the variances in gene alterations in HER2+/HR- breast cancer among Chinese and Western women, we selected patients with HER2+/HR- breast cancer based on the pathological index from the published databases TCGA PanCancer Atlas 15 and METABRIC 16, which included 78 and 110 HER2+/HR- breast cancer cases, respectively (www.cBioportal.org). We compared the mutated gene profiles of our cohort with those from these two databases, and the 25 most frequently mutated genes across the three cohorts are depicted in Figure 2a. For the two most commonly mutated genes, *TP53* and *PIK3CA*, there were no significant differences in mutation rates across the three cohorts (*TP53*: NW China 71.1% vs. TCGA 70.5% vs. METABRIC 79.1%, *p*>0.05; *PIK3CA*: NW China 35.6% vs. TCGA 33.3% vs. METABRIC 38.2%, *p*>0.05). Furthermore, the comparison revealed differences between our cohort and the two published cohorts. Several genes, including *ARID1A*, *PKHD1*, *PTPN13*, *FANCA*, *SETD2*, *BRCA2*, *BLM, STAG2*, *FAT1*, *TOP2A*, *POLE*, *ATM*, *KMT2B*, *FGFR4*, and *EPAS1*, were found to be more frequently mutated in our cohort than in the two published cohorts, suggesting that Chinese women with HER2-positive breast cancer exhibit a distinct gene mutation spectrum compared to Western women.

We also compared the top 10 CNAs among the three HER2-positive breast cancer cohorts (Figure 2b). Statistical results revealed that *ERBB2* was the most frequently amplified gene across all three cohorts (NW China 77.81%, TCGA 70.50%, METABRIC 93.61%), although the *ERBB2* amplification rate in our cohort was lower than that in the METABRIC cohort (*p*=0.009). Regarding the CNAs of the *CDK12* and *MYC* genes, our cohort exhibited a lower rate compared to the METABRIC cohort (*CDK12*: NW China 40.4% vs. METABRIC 79.1%,* p*=0.000; *MYC*: NW China 13.37% vs. METABRIC 39.10%, *p*=0.002).

**Figure 3:**
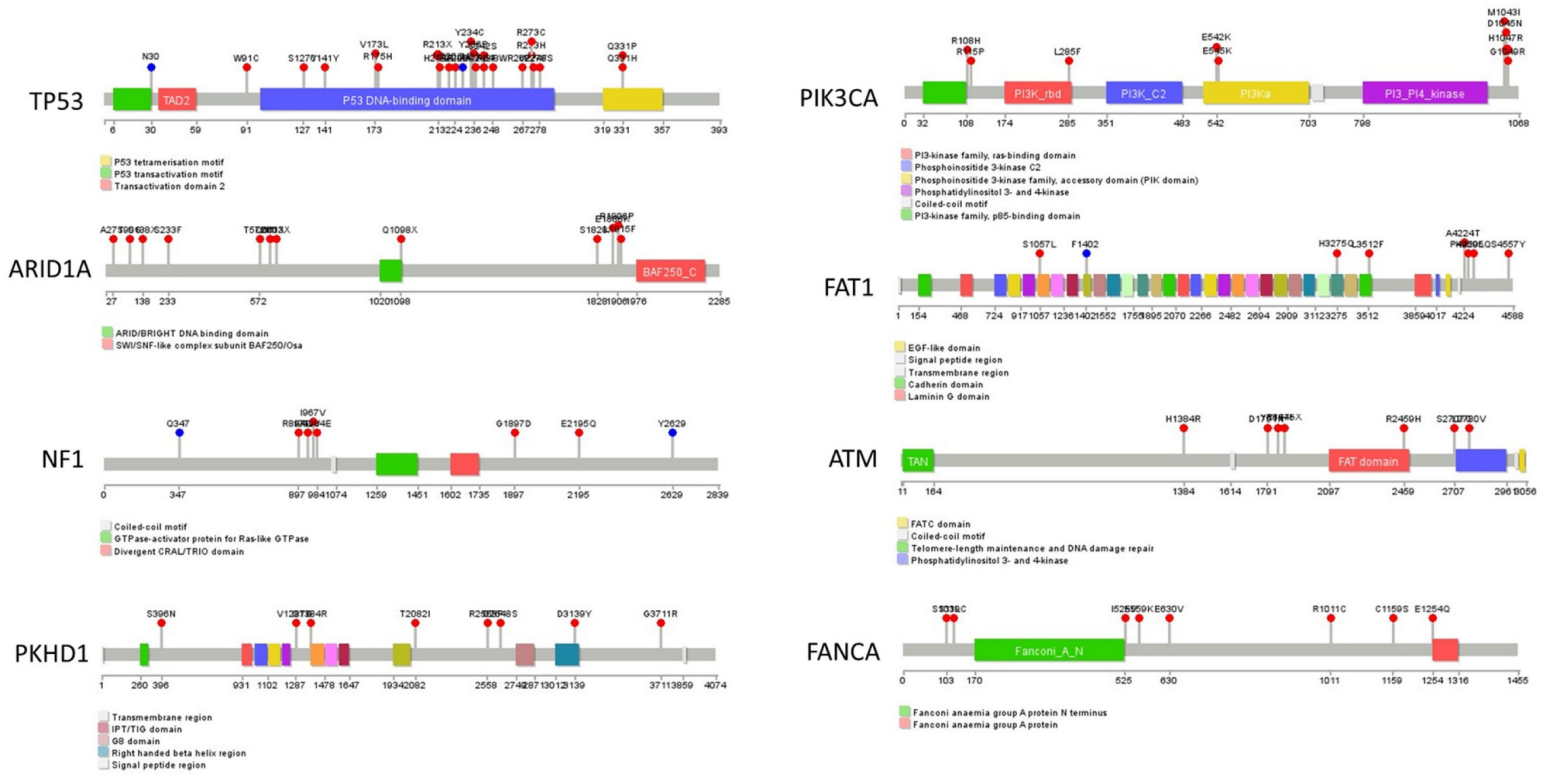
** Summary of hot spot mutations of eight highly mutated genes (Containing *TP53, PIK3CA, ARID1A, FAT1, NF1, ATM, PKHD1*, and* FANCA*) in the HER2+/HR- breast cancer cohort of Northwestern China.** The mutations are shown in the lollipop graphs.

The hotspot mutations in our cohort's top eight mutated genes, including *TP53*, *PIK3CA*, *ARID1A*, *FAT1*, *NF1*, *ATM*, *PKHD1*, and *FANCA*, are illustrated as lollipops in Figure 3. Furthermore, the hotspot sites of the two most mutated genes, *PIK3CA* and *P53*, were compared between our cohort and the two previously mentioned cohorts (Supplementary Figure 1). This comparison revealed that the most frequently mutated site of PIK3CA gene in our cohort, H1047R, was also the most frequently mutated site of PIK3CA in the TCGA and METABRIC cohorts. Additionally, the mutation spectrum of the *TP53* gene in our cohort showed similarities with these two cohorts.

### Pathway enrichment of gene alteration and gene association and mutual exclusion analysis in HER2+/HR- breast cancer

**Figure 4:**
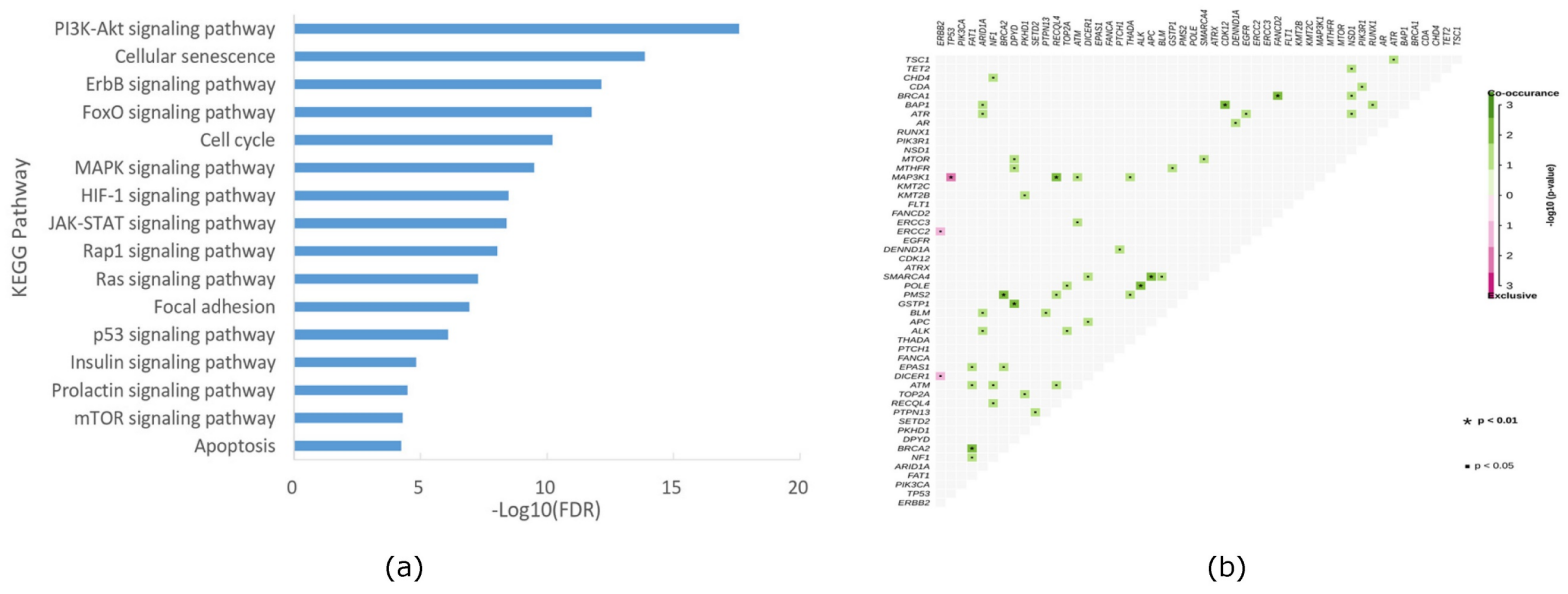
KEGG enrichment of mutation pathway(a) and gene association and mutual exclusion (b) in the HER2+/HR- breast cancer cohort of Northwestern China.

To investigate whether the altered genes in our cohort could be clustered into different pathways related to cancer, we conducted pathway analysis using KEGG. As depicted in Figure 4a, signal transduction-related pathways (including PI3K-AKT, Foxo, ErbB, MAPK, JAK-STAT, HIF-1, RAS, insulin, Hippo, AMPK, and VEGF signaling pathways), cell growth and death-related pathways (including cellular senescence, cell cycle, and apoptosis signaling pathways), and cell movement-related pathways (including adherent junction and focal adhesion signaling pathways) were the predominant enriched pathways. Notably, downstream of the HER2 pathway, PI3K-AKT signaling emerged as the most dominant enriched pathway in our cohort, aligning with previous studies 17. Furthermore, we conducted co-occurrence and mutual exclusion analyses of altered genes in our cohort, with the results presented in Figure 4b.

### Correlation of altered genes with primary trastuzumab resistance in HER2+/HR- breast cancer

**Figure 5:**
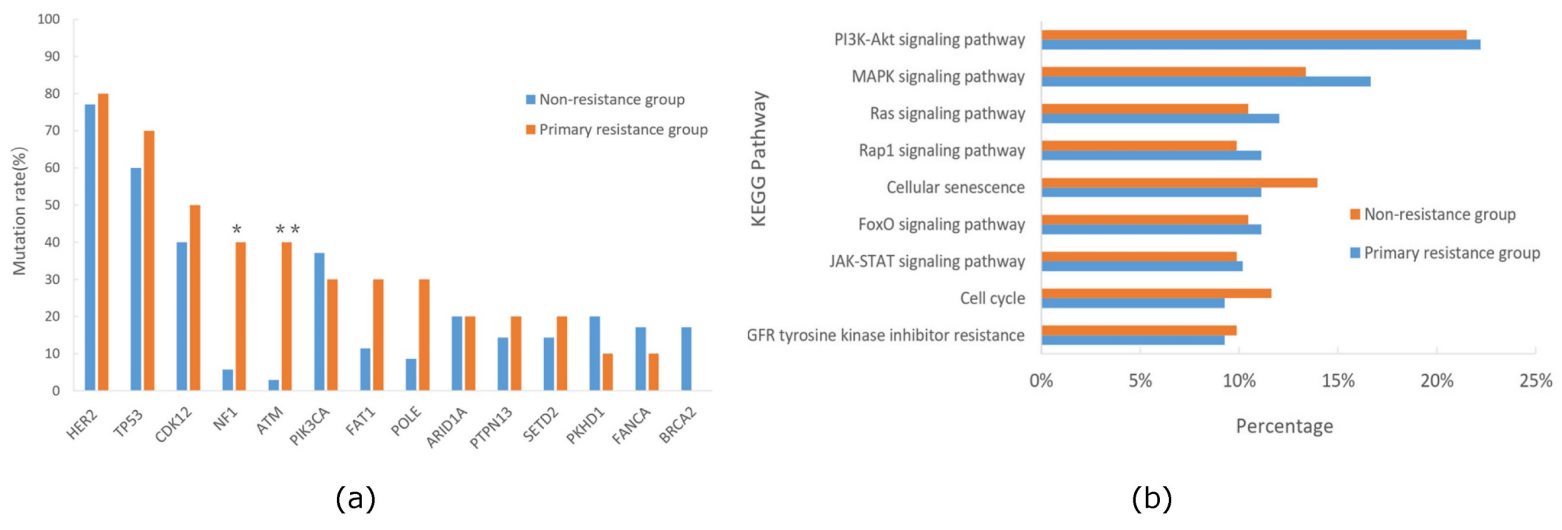
Comparison of mutation genes(a)and KEGG pathway(b) between primary trastuzumab-resistant and non-resistance groups of the HER2+/HR- breast cancer cohort in Northwestern China.

Some patients with HER2-positive breast cancer experienced relapse during or within 12 months after completing 1-year course of trastuzumab treatment, a condition deemed as primary trastuzumab resistance according to prior studies 18-20. All patients in our cohort received standard adjuvant trastuzumab treatment for one-year post-operation, and some patients experienced relapse with a short interval. The follow-up results showed that 22.2% of patients relapsed within 24 months post-operation, suggesting primary resistance to trastuzumab. Consequently, we categorized our cohort into primary trastuzumab-resistant and non-resistant groups based on whether they relapsed within 24 months post-operation, and then compared the gene mutation profiles between these two groups. The analysis indicated significantly higher mutation frequencies of *NF1* and* ATM* in the primary trastuzumab-resistant group compared to the non-resistant group (*NF1*, 40.0% vs. 5.7%, *p*=0.016; *ATM*, 40.0% vs. 2.8%,* p*=0.006), unlike mutations in* PIK3CA* or* ERBB2*, which were associated with trastuzumab resistance in other studies 21-23 (Figure 5a). Additionally, we conducted KEGG pathway enrichment analyses for both groups, revealing the PI3K-AKT signaling pathway as prevalent in both, while the MAPK signaling pathway was more prominent in the primary trastuzumab-resistant group, though without statistical significance (Figure 5b).

To investigate the factors responsible for primary trastuzumab resistance in our cohort, we conducted logistic regression analysis on potential factors, including six clinical characteristics and ten significantly altered genes. The univariable regression analysis revealed that three factors: lymph node stage (*p*=0.042), mutated* NF1* (*p*=0.014), and mutated* ATM* (*p*=0.009), might be associated with trastuzumab resistance. Subsequently, these three factors, along with others with *p*-values <0.2 in the univariable analysis, such as TNM stage, mutated* FAT1*, and mutated* POLE*, were subjected to the multivariable analysis. The analysis determined that only mutated* NF1* (*p*=0.042) and mutated* ATM* (*p*=0.021) were statistically significant (Table 2), suggesting a link between *NF1* and *ATM* mutations and primary trastuzumab resistance in our cohort.

### Analysis of the predictive factor for the recurrence-free survival of HER2+/HR- breast cancer

**Figure 6:**
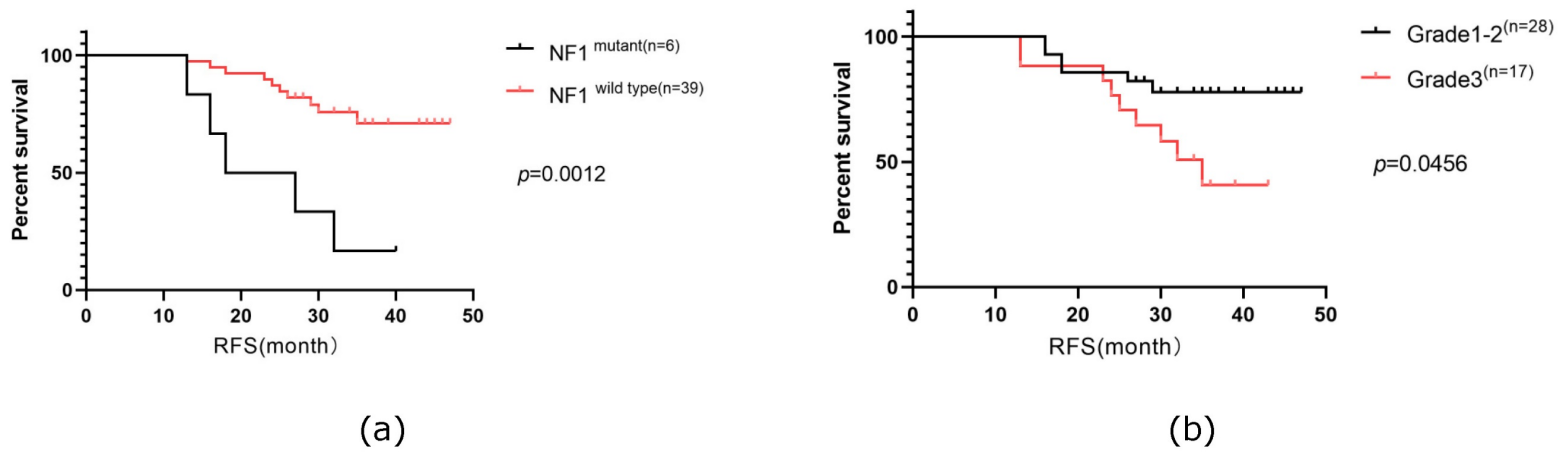
The Kaplan-Meier curves of mutated* NF1* gene (a)and histological grade status(b) on RFS of the patients with HER2+/HR- breast cancer in Northwestern China.

RFS in our cohort was followed up for a median time of 36 months, during which 33.3% of the patients experienced relapse or progression. Consequently, we investigated the prognostic factors for RFS. Our analysis included six clinical characteristics (age, menopausal status, TNM stage, lymph node stage, histologic grade, and Ki67 index) and ten of the most frequently altered genes (*PIK3CA*, *TP53*, *ARID1A*, *FAT1*, *NF1*, *PKHD1*, *ATM*, *FANCA*, *ERBB2*, and *CDK12*) in a Cox univariable regression analysis. This analysis identified four significant factors: histological grade (*p*=0.048), Ki67 index (*p*=0.035), mutated *NF1* (*p*=0.001), and mutated *ATM* (*p*=0.003). These factors, along with three additional factors with *p*-values were <0.2 (TNM stage (*p*=0.136), lymph node stage (*p*=0.159), mutated *ERBB2* (*p*=0.152), were further analyzed in a Cox multivariate regression analysis. Ultimately, mutated *NF1* (*p*=0.009) and histological grade (*p*=0.028) emerged as independent prognostic factors for RFS in patients with HER2+/HR- breast cancer in our cohort (Table 3). Survival curves were generated using the Kaplan-Meier method (Figure 6).

## Discussion

Activation of tumor-associated signaling pathways mediated by the amplified *HER2* gene is involved in 15%-20% of breast cancers 2. Anti-HER2 therapy, including monoclonal antibodies, small molecule kinase inhibitors, and antibody-drug conjugates, has significantly improved clinical outcomes in the HER2-positive subtype of breast cancer. However, some patients with HER2-positive disease still experience progression or relapse during or after anti-HER2 therapy, underscoring the molecular complexity of cancer and highlighting the need to explore the comprehensive molecular profile of HER2-positive breast cancer. In this study, we focus on the HER2+/HR- breast cancer in Northwestern China and aim to investigate the genomic alterations and their correlation with clinical outcomes. We identified 650 genomic alterations in 268 genes in 45 primary breast cancer tissues using a gene-targeted genome sequencing method that included 425 cancer-associated genes. The most frequently mutated genes were *TP53* and *PIK3CA,* and the most frequently amplified genes were *ERBB2*, *CDK12*, and *MYC* in our cohort.

Given that all breast cancer samples in our study were obtained from Chinese women of Asian ethnicity, we were curious about potential differences in the gene mutation spectrum compared to individuals of different ethnic backgrounds. Consequently, we examined the extensive breast cancer mutation data within the TCGA database, securing 78 and 110 HER2+/HR- breast cancer cases from the PanCancer Atlas and METABRIC datasets, respectively. In comparing the top 20 mutated genes across the three cohorts, we observed that *TP53* and *PIK3CA* remained the most frequently mutated genes in all datasets, with no significant statistical difference in mutation rates. However, our cohort exhibited a notably higher mutation rate in the *FANCA*, *ARID1A*, *SETD2*, *BRCA2*, *BLM*, *FAT1*, *TOP2A*, *PKHD1*, *PTPN13*, *POLE*, *ATM*, and *KMT2B* genes compared to the other two cohorts (Figure 2). Among these genes, several are recognized as tumor-related genes critical to the onset and progression of breast cancer, including *FANCA*, *ARID1A*, *BRCA2*, *BLM*, *TOP2A*, *ATM*, and *KMT2B* 24-30. Additionally, we noted that the mutation rate in these genes exceeded 10%, a rarity in other published breast cancer mutation databases 31-33. This suggests that HER2+/HR- breast cancer in Chinese women presents a distinct mutation spectrum from that observed in Western populations.

Although the clinical application of anti-HER2 drugs has markedly improved the survival for patients with HER2-positive breast cancer, a subset of these patients still experiences treatment failure at some stage. This failure is often attributed to primary and acquired resistance for anti-HER2 therapy. Recent researches 17, 22, 23, 34-37 suggest that anti-HER2 resistance often stems from intratumor heterogeneity, specific HER2 splicing variants, activation of the PIK3CA/AKT signaling pathway, signaling pathway crosstalk, or cell cycle sensitization.

Our study focus on patients with HER2+/HR- breast cancer, identifying a subset that experienced recurrence during or within one year following trastuzumab treatment, typically classified as primary trastuzumab resistance 20. Additionally, we investigated genetic factors that might underlie primary trastuzumab resistance in our cohort. We discovered that mutations in the *ATM* and *NF1* tumor suppressor genes were significantly more common in the primary trastuzumab-resistant group compared to the non-resistant group (*ATM*, 40.0% vs. 2.8%*, p*=0.006; *NF1*, 40.0% vs. 5.7%, *p*=0.016). Furthermore, these mutations in *ATM* and *NF1* were associated with primary trastuzumab resistance in both univariate and multivariate regression analyses (Table 2).

*ATM*, the gene mutated in ataxia-telangiectasia (AT), regulates the DNA damage response by playing a pivotal role in identifying and repairing double-strand breaks, thereby being linked to various cancer-related processes such as tumorigenesis, progression, metabolism, drug resistance, and radiosensitivity 38-40. Moslemi *et al.* 41 found that somatic ATM missense mutations are associated with a 2.8- to 3-fold increased risk of sporadic breast cancer. In addition, clinical data analysis revealed that breast cancers harboring *ATM* mutations are highly aggressive and exhibit reduced progression-free survival and overall survival 42. A recent study demonstrated that *ATM* can maintain HER2 protein stability by forming a complex with HER2 and HSP90, thus inhibiting HER2 ubiquitination and degradation in HER2-positive breast cancer cell lines 29. In our research, *ATM* mutations were linked to primary trastuzumab resistance in patients with HER2+/HR- breast cancer, indicating that *ATM* is involved in the anti-HER2 resistance and the underlying mechanism is worth studying further in the future.

*NF1*, which encodes neurofibromin and negatively regulates Ras activation by converting GTP-Ras to GDP-Ras, acts as a tumor suppressor gene 43. The loss of neurofibromin due to *NF1* mutation or deletion leads to the activation of Ras-Raf-MEK-ERK/Ras-PI3K-Akt signaling pathways, playing a significant role in the initiation and progression of various tumors 44-46. Somatic mutations of *NF1* are frequently observed in many tumors, correlating with tumorigenesis and progression 44, 47-50. In breast cancer, *NF1* mutations are commonly identified through large-scale sequencing, and these mutations are associated with endocrine therapy resistance and poor outcomes 51-53. Recent studies have shown that *NF1* mutation can induce acquired resistance to anti-HER2 therapy in HER2-positive breast cancer. It has been demonstrated that the loss of NF1, mediated by mutation, enables HER2-positive breast cancer to adapt to HER2-targeted treatment by shifting from PI3K/AKT signaling pathway to MAPK signaling pathway 54-56. In our study, we found that mutated *NF1* was correlated with primary trastuzumab resistance and poor RFS in patients with HER2+/HR- breast cancer in Northwest China. Additionally, we conducted pathway enrichment analysis and identified a predominant enrichment of mutations in the MAPK signaling pathway among the primary trastuzumab-resistant patient group. Taken together, these alterations suggest a direct contribution to anti-HER2 resistance, warranting further investigation into the underlying mechanisms.

While our study has yielded some notable results, it is not devoid of limitations. First, the small number of patients enrolled limits the representativeness of the mutation spectrum of HER2+/HR- breast cancer in China. Second, our cohort's follow-up period, which spans only three years of relapse-free survival data, is insufficient for long-term analysis, limiting the prognostic value of the mutated genes identified in our cohorts. Third, the relationship between mutated genes and primary trastuzumab resistance in patients with HER2+/HR- breast cancer in China necessitates further exploration.

## Conclusions

In conclusion, our study revealed a distinct mutation landscape in Northwestern Chinese patients with HER2+/HR- breast cancer, differing from the mutation profiles reported in Western HER2+/HR- breast cancer cohorts. Additionally, our study identified mutated genes associated with primary trastuzumab resistance and RSF in patients with HER2+/HR- breast cancer, which offers insights into improving the efficacy of anti-HER2 therapy for patients with HER2+/HR- breast cancer in China.

## Supplementary Material

Supplementary figure and table.

## Figures and Tables

**Table 1:** Clinicopathological features of the HER2+/HR- breast cancer cohort in Northwestern China.

Clinicopathological features	Total cohort, n=45 (%)
Age (median, range)	53.0 (26-80)
Menopausal status	
Premenopausal	17 (37.8%)
Postmenopausal	28 (62.2%)
pTNM stage	
I stage	0 (0.0%)
II stage	32 (71.1%)
III stage	13 (28.9%)
Lymph node stage	
N0	0 (0.0%)
N1	34 (75.5%)
N2	11 (24.5%)
N3	0 (0.0%)
Histological grade	
1	0 (0.0%)
2	28 (62.2%)
3	17 (37.8%)
Ki 67 index	
≤30%	19 (42.2%)
>30%	26 (57.8%)
Recurrence	
Yes	15 (33.3%)
RFS≤24 months	10 (22.2%)
RFS>24 months	5 (11.1%)
No	30 (66.7%)

**Table 2:** Binary logistic regression analysis of factors related to primary trastuzumab resistance in the HER2+/HR- breast cancer cohort of Northwestern China.

Parameter	Univariable analysis			Multivariable analysis		
	HR	95% CI	*p-*value	HR	95% CI	*p-*value
TNM stage III vs.I-II	3.375	0.776 to14.669	0.105			
Lymph node stage N2-N3 vs. N0-N1	4.833	1.057 to 22.091	0.042			
FAT1 gene Mutation vs. WT	3.321	0.603 to 18.307	0.168			
NF1 gene Mutation vs. WT	11.000	1.633 to 74.083	0.014	9.004	1.078 to 75.192	0.042
ATM gene Mutation vs. WT	22.667	2.147 to 239.32	0.009	18.919	1.599 to 229.630	0.021
POLE gene Mutation vs. WT	4.571	0.758 to 27.577	0.097			

**Table 3:** Univariable and multivariable analysis of factors related to the RFS of the patients with HER2+/HR- breast cancer in Northwestern China.

Parameter	Univariable analysis			Multivariable analysis		
	HR	95% CI	*p-*value	HR	95% CI	*p-*value
pTNM stage III vs. I-II	2.201	0.780 to 6.209	0.136			
Lymph node stage N2-3 vs. N0-1	2.170	0.738 to 6.382	0.159			
Histological grade 3 vs. 1-2	2.835	1.007 to 7.980	0.048	3.613	1.145 to 11.394	0.028
Ki67 index High vs. Low-Medium	3.907	1.099 to 13.887	0.035			
NF1 gene Mutation vs. WT	6.270	2.096 to 18.758	0.001	5.965	1.550 to 22.957	0.009
ATM gene Mutation vs. WT	5.961	1.826 to 19.455	0.003			
HER2 gene Mutation vs. WT	2.534	0.709 to 9.054	0.152			
