# Supplementary material for: Genomic Alterations Correlated to Trastuzumab Resistance and Clinical Outcomes in HER2+/HR- Breast Cancers of Patients Living in Northwestern China
Source: J Cancer. 2024 Jun 17;15(14):4467–76. doi: 10.7150/jca.84832 (PMC11242333; doi:10.7150/jca.84832)
Supplement: Supplementary file 1 — Supplementary figure and table. [file jcav15p4467s1.pdf]

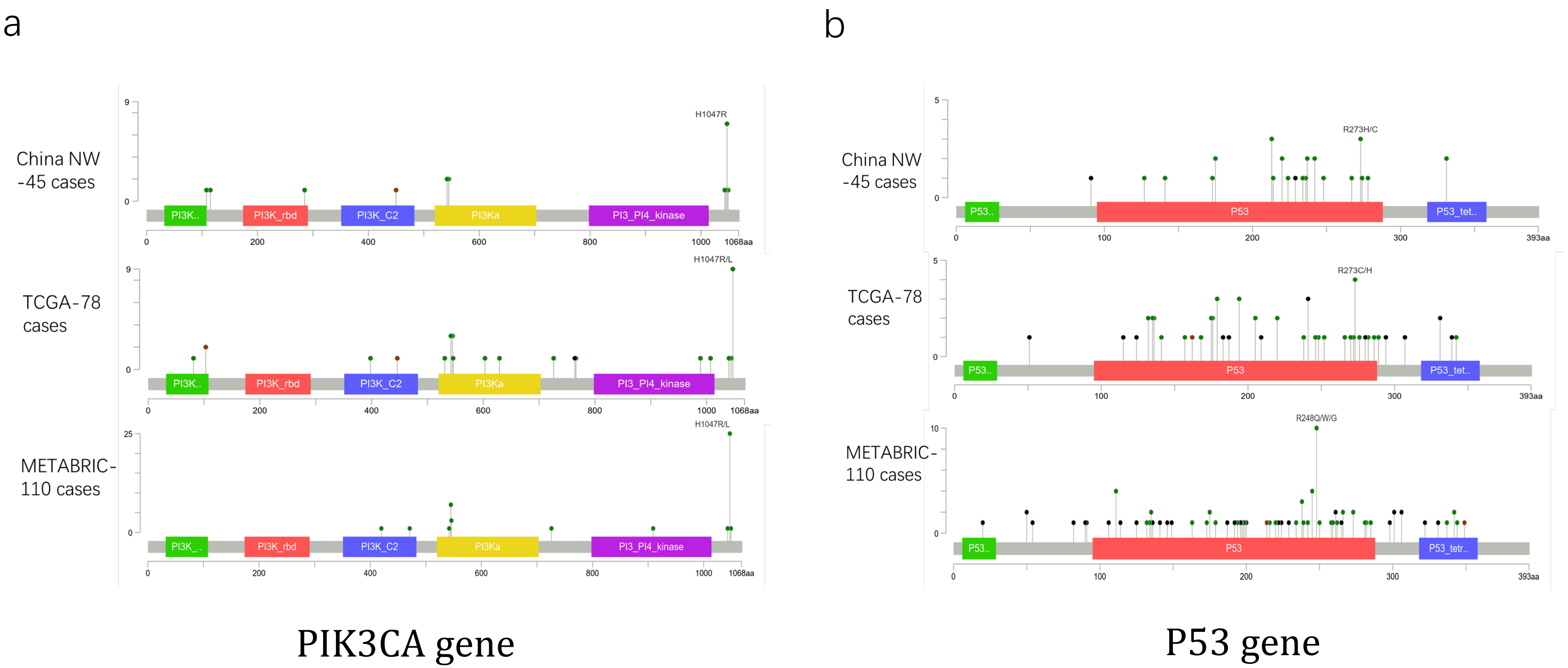


**Supplement Figure 1** Comparison of hot spot mutation of PIK3CA (a) and P53 (b)gene in the three HER2+/HR− breast cancer cohorts

Table S1. 425 key cancer related genes in our Geneseeq pan-cancer gene panel.

| | | | | | | |
|---|---|---|---|---|---|---|
| ABCB1(MDR | CDC73 | ERCC2 | IDH2 | MTOR | PRKACA | SOX14 |
| ABCB4 | CDH1 | ERCC3 | IFNG | MUTYH | PRKACG | SOX2 |
| ABCC2(MRP | CDK10 | ERCC4 | IFNGR1 | MYC | PRKAR1 | SOX21 |
| ADH1A | CDK12 | ERCC5 | IGF1R | MYCL | PRKCI | SPOP |
| ADH1B | CDK4 | ESR1 | IGF2 | MYCN | PRKDC | SPRY4 |
| ADH1C | CDK6 | ETV1 | IKBKE | MYD88 | PRSS1 | SRC |
| AIP | CDK8 | ETV4 | IKZF1 | MYH9 | PRSS3 | SRY |
| AKT1 | CDKN1A | ETV6 | IL7R | NAT1 | PTCH1 | STAG2 |
| AKT2 | CDKN1B | EWSR1 | INPP4B | NBN | PTEN | STAT3 |
| AKT3 | CDKN1C | SPOP | IRF2 | NCOR1 | PTK2 | STK11 |
| ALDH2 | CDKN2A | EXT2 | JAK1 | NF1 | PTPN11 | STMN1 |
| ALK | CDKN2B | EZH2 | JAK2 | NF2 | PTPN13 | STT3A |
| AMER1 | CDKN2C | FANCA | JAK3 | NFE2L2 | PTPRD | SUFU |
| APC | CEBPA | FANCC | JARID2 | NFKBIA | QKI | TAP1 |
| AR | CEP57 | FANCD2 | JUN | NKX2-1 | RAC1 | TAP2 |
| ARAF | CHD4 | FANCE | KDM5A | NKX2-4 | RAC3 | TEK |
| ARID1A | CHEK1 | FANCF | KDM6A | NOTCH1 | RAD50 | TEKT4 |
| ARID1B | CHEK2 | FANCG | KDR(VEGF | NOTCH2 | RAD51 | TERC |
| ARID2 | CREBBP | FANCI | KEAP1 | NOTCH3 | RAD51B | TERT |
| ARID5B | CRKL | FANCL | KIF1B | NPM1 | RAD51C | TET2 |
| ASCL4 | CSF1R | FANCM | KIF5B | NQO1 | RAD51D | TGFBR2 |
| ASXL1 | CTCF | FAT1 | KIT | NRAS | RAD54L | THADA |
| ATF1 | CTLA4 | FBXW7 | KITLG | NRG1 | RAF1 | TMEM127 |
| ATIC | CTNNB1 | FGF19 | KLLN | NSD1 | RARA | TMPRSS2 |
| ATM | CUL3 | FGFR1 | KMT2A(M | NTRK1 | RARG | TNFAIP3 |
| ATR | CUX1 | FGFR2 | KMT2B | NTRK2 | RASGEF1 | TNFRSF11A |
| ATRX | CXCR4 | FGFR3 | KMT2C | NTRK3 | RB1 | TNFRSF14 |
| AURKA | CYLD | FGFR4 | KMT2D(M | PAK3 | RECQL4 | TNFRSF19 |
| AURKB | CYP19A1 | FH | KRAS | PALB2 | RELN | TNFSF11 |
| AXIN2 | CYP2A13 | FLCN | LHCGR | PALLD | RET | TOP1 |
| AXL | CYP2A6 | FLT1(VEGFR | LMO1 | PARK2 | RHOA | TOP2A |
| B2M | CYP2A7 | FLT3 | LRP1B | PARP1 | RICTOR | TP53 |
| BAD | CYP2B6*6 | FLT4 | LYN | PARP2 | RNF43 | TP63 |
| BAI3 | CYP2C19*2 | FOXA1 | LZTR1 | PAX5 | ROS1 | TPMT |
| BAK1 | CYP2C9*3 | FOXP1 | MAP2K1 | PBRM1 | RPTOR | TSC1 |
| BAP1 | CYP2D6 | FRG1 | MAP2K2 | PDCD1(PD | RRM1 | TSC2 |
| BARD1 | CYP3A4*4 | GATA1 | MAP2K4 | PDCD1LG2 | RUNX1 | TSHR |

| | | | | | | |
|---|---|---|---|---|---|---|
| BAX | CYP3A5 | GATA2 | MAP3K1 | PDE11A | RUNX1T | TTF1 |
| BCL2 | DAXX | GATA3 | MAP3K4 | PDGFRA | SBDS | TUBB3 |
| BCL2L11(BI | DDR2 | GATA4 | MAP4K3 | PDGFRB | SDC4 | TUBB4A |
| BCR | DENND1A | GATA6 | MAX | PDK1 | SDHA | TUBB4B |
| BIRC3 | DHFR | GNA11 | MCL1 | PGR | SDHB | TUBB6 |
| BLM | DICER1 | GNAQ | MDM2 | PHOX2B | SDHC | TYMS |
| BMPR1A | DLL3 | GNAS | MDM4 | PIK3C3 | SDHD | U2AF1 |
| BRAF | DNMT3A | GRIN2A | MECOM | PIK3CA | SEPT9 | UGT1A1 |
| BRCA1 | DPYD | GRM3 | MED12 | PIK3R1 | SETBP1 | VAMP2 |
| BRCA2 | DUSP2 | GRM8 | MEF2B | PIK3R2 | SETD2 | VEGFA |
| BRD4 | EGFR | GSTM1 | MEN1 | PKHD1 | SF3B1 | VHL |
| BRIP1 | EML4 | GSTM4 | MET | PLAG1 | SGK1 | WAS |
| BTG2 | EP300 | GSTM5 | MGMT | PLK1 | SLC34A2 | WISP3 |
| BTK | EPAS1 | GSTP1 | MITF | PMS1 | SLC3A2 | WRN |
| BUB1B | EPCAM | GSTT1 | MLH1 | PMS2 | SLC7A8 | WT1 |
| c11orf30 | EPHA2 | HDAC2 | MLH3 | POLD1 | SMAD2 | XPA |
| CASP8 | EPHA3 | HDAC9 | MLLT1 | POLD3 | SMAD3 | XPC |
| CBL | EPHA5 | HGF | MLLT3 | POLE | SMAD4 | XRCC1 |
| CBLB | EPHB2 | HLA-A | MLLT4 | POLH | SMAD7 | YAP1 |
| CCND1 | ERBB2(HE | HNF1A | MPL | POT1 | SMARCA | ZNF2 |
| CCNE1 | ERBB2IP | HNF1B | MRE11A | PPARD | SMARCB | ZNF217 |
| CD274(PD- | ERBB3 | HRAS | MSH2 | PPP2R1A | SMO | ZNF703 |
| CD74 | ERBB4 | HSD3B1 | MSH6 | PRDM1 | SOS1 | |
| CDA | ERCC1 | IDH1 | MTHFR | PRF1 | SOX1 | |
